# TME-targeted approaches of brain metastases and its clinical therapeutic evidence

**DOI:** 10.3389/fimmu.2023.1131874

**Published:** 2023-05-09

**Authors:** Ibrar Muhammad Khan, Safir Ullah Khan, Hari Siva Sai Sala, Munir Ullah Khan, Muhammad Azhar Ud Din, Samiullah Khan, Syed Shams ul Hassan, Nazir Muhammad Khan, Yong Liu

**Affiliations:** ^1^ Anhui Province Key Laboratory of Embryo Development and Reproduction Regulation, Anhui Province Key Laboratory of Environmental Hormone and Reproduction, School of Biological and Food Engineering, Fuyang Normal University, Fuyang, China; ^2^ Hefei National Laboratory for Physical Sciences at the Microscale, School of Life Sciences, University of Science and Technology of China, Hefei, China; ^3^ MOE Key Laboratory of Macromolecular Synthesis and Functionalization, International Research Center for X Polymers, Department of Polymer Science and Engineering, Zhejiang University, Hangzhou, China; ^4^ Faculty of Pharmacy, Gomal University, Dera Ismail Khan, KPK, Pakistan; ^5^ Institute of Entomology, Guizhou University, Scientific Observing and Experimental Station of Crop Pests, Guiyang, Ministry of Agricultural and Affairs, Guiyang, China; ^6^ Department of Natural Product Chemistry, School of Pharmacy, Shanghai Jiao Tong University, Shanghai, China; ^7^ Department of Zoology, University of Science and Technology, Bannu, Pakistan

**Keywords:** brain metastases, tumor microenvironment, central nervous system barrier, immunotherapy, molecular mechanism

## Abstract

The tumor microenvironment (TME), which includes both cellular and non-cellular elements, is now recognized as one of the major regulators of the development of primary tumors, the metastasis of which occurs to specific organs, and the response to therapy. Development of immunotherapy and targeted therapies have increased knowledge of cancer-related inflammation Since the blood-brain barrier (BBB) and blood-cerebrospinal fluid barrier (BCB) limit immune cells from entering from the periphery, it has long been considered an immunological refuge. Thus, tumor cells that make their way “to the brain were believed to be protected from the body’s normal mechanisms of monitoring and eliminating them. In this process, the microenvironment and tumor cells at different stages interact and depend on each other to form the basis of the evolution of tumor brain metastases. This paper focuses on the pathogenesis, microenvironmental changes, and new treatment methods of different types of brain metastases. Through the systematic review and summary from macro to micro, the occurrence and development rules and key driving factors of the disease are revealed, and the clinical precision medicine of brain metastases is comprehensively promoted. Recent research has shed light on the potential of TME-targeted and potential treatments for treating Brain metastases, and we’ll use that knowledge to discuss the advantages and disadvantages of these approaches.

## 1 Introduction

Metastasis occurs when cancer cells divide and spread from the main tumor to other parts of the body *via* the circulatory or lymphatic systems (1). 90% of cancer-related fatalities are caused by metastasis (2, 3). In particular, brain metastasis is a significant problem that often results in terrible effects for the patient (4). Some tumors have a propensity to colonize specific organs like the brain, which presents a significant challenge in studying the biology of metastasis. Priority organ tropism is mediated by genetic markers that have been found (5). The components that help tumor cells get past tissue-specific barriers (such the blood-brain barrier) or create cancer permit-niches in possibly hostile environments are typically linked to gene expression differences in tumor cell types with strong organ-specific tropism (6, 7). The ability of tumor cells to rapidly absorb niches cells in foreign systems for their function as well as to suppress or evade anti-tumor activity determines the success of metastatic colonization in addition to the tumor cells’ inherent characteristics.

Upon entering the central nervous system (CNS), Tumor cells are greeted with a vastly different cellular and matrix structure, metabolism, and immunological milieu than they encountered in the primary site (8, 9). In addition to neurons cells, the brain also contains astrocytes, oligodendrocytes and microglia, which support the brain’s normal functioning. Recent research has focused on immune and inflammatory cells generated from the blood as significant mediators of inflammation linked with brain metastases and cell types already known to reside in the brain (10). Tumor-infiltrating lymphocytes indicate a favorable prognosis and response to immunotherapy; nevertheless, many myeloid cells are linked to immunosuppression, tumor development, and treatment resistance (11).

Brain metastases are an important cause of treatment failure and death in cancer patients (12). As one of the most common tumor metastasis targets, the brain has an extremely complex anatomical structure, diverse cell types, and important physiological functions (13). Tumor cells in the blood circulation are transferred to various regions in the brain in multiple ways, and the local microenvironment conditions are different. The weak CNS barrier and the considerable geographic variability of brain metastases make successful therapeutic therapies challenging (14, 15).Thus, it is important to investigate the signaling pathway of the tumor microenvironment in depth and actively seek new therapeutic targets according to the initial formation of distinct brain metastases, so as to offer a fresh viewpoint on how to improve the prognosis of patients with new therapeutic approaches of brain metastases (16).This review sheds insight into the intricate relationship between tumor cells and the niche cells surrounding tumors. We also go through the current state of our understanding of the tumor microenvironment’s (TME) cell type-specific precursors’ anticancer role in brain metastases (BrM). Using this research, we will examine the potential and limitations of TME-targeted immunotherapies for brain metastases.

## 2 Brain metastasis diagnosis

According to the location of metastasis, central nervous system metastases can be divided into brain parenchymal metastasis (BrM), leptomeningeal metastasis (LM), and dural metastasis (dural metastasis DM), there were significant epidemiological differences among different metastases. Parenchymal brain metastases are the most general type of central nervous system metastases, mainly from hematogenous spread, common in the middle cerebral artery distribution of the gray matter junction and the arterial circulatory junction between the middle cerebral artery and the posterior cerebral artery. In solid tumors, the incidence of BrM is 20%-40%, and its incidence is 10 times that of primary malignant brain tumors (17). The most common primary types of BrM are breast cancer (15-30%), melanoma (5-20%),lung cancer (40-50%), and rectal cancer (3-8%) (18), and the median survival time is generally 3-6 months. LM is a complication of cancer in which tumor cells diffuse into the cerebrospinal fluid (CSF) and subarachnoid space to form multifocal or diffuse growth. LM generally occurs in the late stage of the disease. Breast, lung, and melanoma are the most common primary tumors leading to LM. In breast cancer and lung cancer patients, the incidence of LM is 5% to 20%. Given the lack (19)of specificity in clinical presentation and the short survival time (4 to 8 weeks), it is difficult to accurately determine the incidence of LM in the population.

Therefore, the incidence of LM is greatly underestimated. DM lesions are mainly located in the epidural space, and the incidence of DM in cancer patients is 9%. Breast and prostate cancer are the two most common primary cancers leading to DM (20). It has been reported that DM tends to have bone metastasis and is easier to colonize in the dural environment close to the cranial bone. Some patients with dural metastasis are complicated with BrM or LM (21). The median survival time of DM patients is about 6 months, and the onset of DM is dangerous. It has not received enough attention in clinical practice, and there is a lack of effective treatment methods and animal model studies.

Furthermore, the difficulties of acquiring intracranial tissue make deciphering the molecular pathways behind brain metastases more challenging (22). In order to guide therapeutic treatment, it is urgently necessary to look at the immunological environment of brain metastases. In recent years, with advances in neuroimaging and the development of new cancer therapies, more effective clinical interventions have prolonged the overall survival of patients with primary tumors. The risk of central nervous system metastasis of tumors is increasing year by year, and the prevention and control of brain metastases should be paid enough attention to in clinical and basic research (23).

## 3 Molecular mechanism of brain metastasis

Most intracranial tumors are brain metastases, originating most frequently in lung cancer (24). NSCLC (non-small cell lung cancer) metastatic brain disease makes up about half of all cases of metastases to the brain.Brain metastases will appear at some time in the disease course in about one-third of NSCLC patients (25). Current therapy options for NSCLC brain metastases are ineffective due to the unique architectural and physiological characteristics of the central nervous system, and the prognosis is dismal (CNS). Another drawback is the dearth of comprehensive studies on brain metastases in NSCLC. Immunotherapy has had a rapid uptake in the treatment of non-small-cell lung cancer (NSCLC) (26, 27). Results from preliminary clinical studies suggest that immune checkpoint inhibitors may benefit certain patients with advanced non-small cell lung cancer (NSCLC) (28). However, because of genetic variations between brain metastases and original tumors and variances in the tumor microenvironment, intracranial and extracranial lesions may react differently to systemic immunotherapy.

NSCLC metastases frequently and preferentially spread to the neurological system (29). Capillary endothelial cells produce cytokines in response to CTCs (Circulating tumor cells) when they pass through the brain’s capillaries slower than the blood. Brain metastases from when tumor cells with high invasive potential travel through the circulatory system, the brain’s lymphatic system, or the cerebrospinal fluid. Once there, they establish themselves in the brain parenchyma, the leptomeninges, or the epidural region (30).

Up to 30% of breast cancer patients whose disease has progressed to other organs, as shown by autopsy, have BM (31). In addition to this, there is evidence to support the contention that the incidence of brain abscesses caused by breast cancer is on the rise (32, 33). According to the prognosis index, people who were diagnosed with breast cancer BM had the best chances of surviving the disease (median OS, 13.8 months) (34);. Researchers have identified a variety of oncogenes that are associated with breast cancer (35). Breast cancer People who have breast cancer that is positive for human epidermal growth factor receptor 2 (HER2) have the highest risk of developing breast hyperplasia, followed by those who have endovascular breast cancer and those who have triple-negative breast cancer (TNBC) (36). Patients who have metastatic HER2-positive breast cancer have a risk of acquiring breast metastases that is two to four times higher than the risk that persons who have breast cancer but no HER2 mutations (37).

Among the several barriers present in the central nervous system, the blood-brain barrier (BBB) and the blood-cerebrospinals fluid barrier (BCSFB) are among the most essential (BTB) (38). In the healthy brain, the initial CNS gatekeepers were the BBB and BCSFB. Protection of the central nervous system against inflammatory injury is achieved by capillary endothelial cells forming tight connections with adjacent connective tissue (brain edema) (39). The spinal cord and brain are not entirely spared by the immune system. For the larger part of the last century, researchers have considered the brain to be a special organ in terms of immunity due to the presence of the blood-brain barrier and the blood-cerebrospinal fluid barrier (40). The discovery of the meningeal lymphatic vessels and the lymphatic system in the brain, however, completely disproved this theory (41). Furthermore, experimental evidence demonstrates that brain metastases contain T lymphocytes and other immune cells from circulation (42). A connection exists between immune cells carried by the blood and brain-based immunological components (43). To reach the deep cervical lymph nodes, particularly immune cells of the central nervous system must first enter the cerebrospinal fluid *via* the endolymph system, then travel through the olfactory bulb, olfactory neuron, lamina Lacrimosa, and the nasal mucosa. However, immune cells can still enter the CNS *via* the hyaline tapetum and lymphatic capillaries in the cerebrospinal fluid. Additionally, CD4-positive memory cells and macrophages T cells play a crucial role in immunological surveillance in the central nervous system (44). They can be found in the ventricles, peripheral nerves, and perivascular spaces.

**Figure 1:**
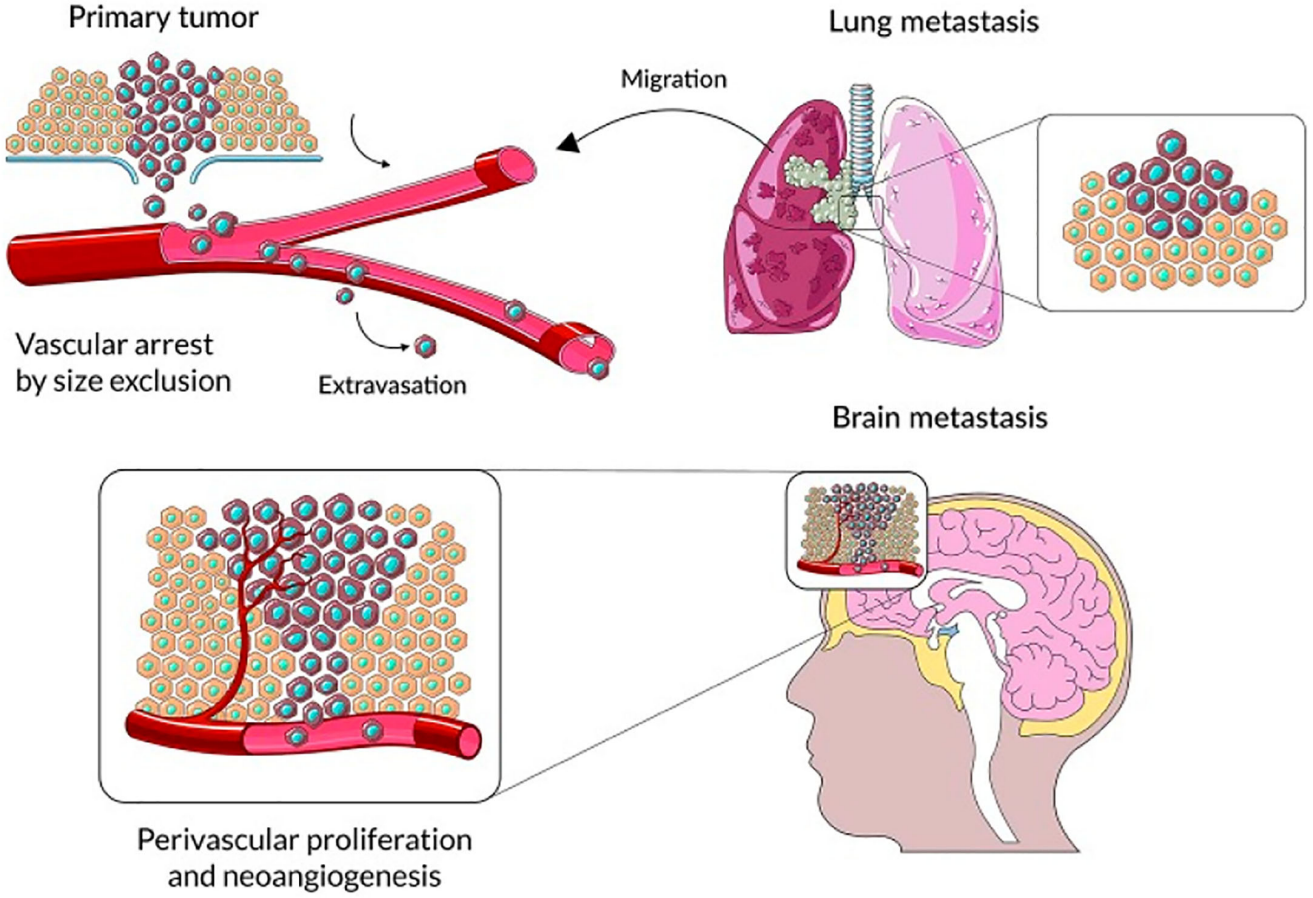
The primary steps in the progression of brain parenchyma cancer cell colonization. Reproduced under common creative licenses from (48).

Numerous malignancies contain tumor-associated macrophages (TAMs), and the actions of stromal cells in the tumor microenvironment suggest that TAMs stimulate a variety of inflammatory and wound-healing processes (45–47). Three primary functional classes of TAMs have been established, each of which performs a particular role. Macrophages called perivascular macrophages are found in the perivascular niche, which is located around blood vessels. These macrophages help tumor cells invade blood arteries and proliferate throughout the body, which in turn promotes tumor angiogenesis **(** **Figure 1**). It is possible that TAMs in circulation will migrate together with cancer cells to a migration and proliferation niche, where they will promote matrix remodeling, tumor progression, and the development of a suppressive microenvironment. A third set of TAMs accumulates in a pre-metastatic niche and helps tumor cells spread extravasatively, seed lesions, and grow them into metastatic lesions. Tumor-associated macrophages (TAMs) disrupt surrounding tissues, inhibit the immune system locally and systemically, and may help tumor cells withstand cytotoxic chemotherapy (49). Tumor microenvironment (TME) stromal cells, in contrast to tumor cells, are genetically stable, making them an appealing target for therapeutic methods because they are not likely to develop drug resistance or lead to tumor recurrence. Researchers Joyce et al. have compiled a wealth of information about the immunological landscape, which they say can shed light on how we might circumvent the TME’s tumor-promoting characteristics and instead use it to our advantage in the battle against cancer (50).

## 4 The main pathways of tumor cell invasion into the central nervous system

Despite the presence of blood brain barrier, blood-derived cancer cells can still infiltrate the nervous system in a variety of ways (51). The blood-brain barrier (BBB) comprises endothelial cells and parenchymal nerve cells. As the main barrier structure for central-peripheral material exchange, BBB helps the CNS to actively absorb nutrients, block the entry of harmful substances, and ensure the normal physiological homeostasis of the CNS (52). In the process of brain metastasis, tumor expansion, vascular heterogeneity, BBB barrier structure damage. Tumor cells in the proliferative stage selectively absorb nutrients through BBB, maintain a highly active metabolic level, and further damage the normal function of blood vessels and neurons (53). The neuropeptide substance P(SP) secreted by breast cancer cells can induce the expression of TNF-α and Ang-2 in human microvascular endothelial cells. Severely affects zonula occludens-1 (zonula occludens-1), which constitutes a blood-brain barrier. ZO-1 localization and distribution and claudin-5 structural changes, thereby increasing the permeability of the BBB (54). In addition, serine proteases released by melanoma cells can degrade the interendothelial bonding complex (55), Additionally, the deepness to which cancer cells invade the central nervous system is considerably increased by the high expression of heparanase during the process of brain metastasis. From the tumor tissues of individuals with advanced breast cancer, researchers extracted cells with a great propensity to spread to the brain (56). By analyzing these cells and clinical samples, COX2, EGFR ligand HBEGF, α2, 6-xylosyl transferase, and α2, 6-sialic acid transferase ST6GALNAC5 were specifically upregulated during brain metastasis to promote tumor cell crossing the BBB.

Tumor cells can also break through the blood CSF barrier (BCSFB) and enter the cerebrospinal fluid circulation, forming distal meningeal metastases (57). Recent reports have shown that acute lymphoblastic leukemia (ALL) is characterized by central nervous system metastasis. Tumor cells can migrate to the pia mater through vertebrae, calf bone marrow, and blood vessels in the subarachnoid space but rarely involve solid neural tissues (58). In addition, the Batson plexus, spinal nerve plexus, and cranial nerve plexus can provide direct pathways for tumor cells to enter the nervous system from the periphery. However, in-depth mechanism research on this method is still lacking at present. Dural metastasis generally comes from the direct extension of skull metastasis (such as advanced nasopharyngeal cancer) or hematogenous metastasis (59). In special cases, it can also be compressed to the dura and surrounding tissues by solid metastasis. Although the epidural annulus is thought to be isolated from the central nervous system, with the growth of epidural metastases, the tumor compresses adjacent blood vessels, nerve roots, and spinal cord, leading to local pain, radiculopathy, and spinal cord disease (60).

## 5 Microenvironment characteristics of brain metastases

**Figure 2:**
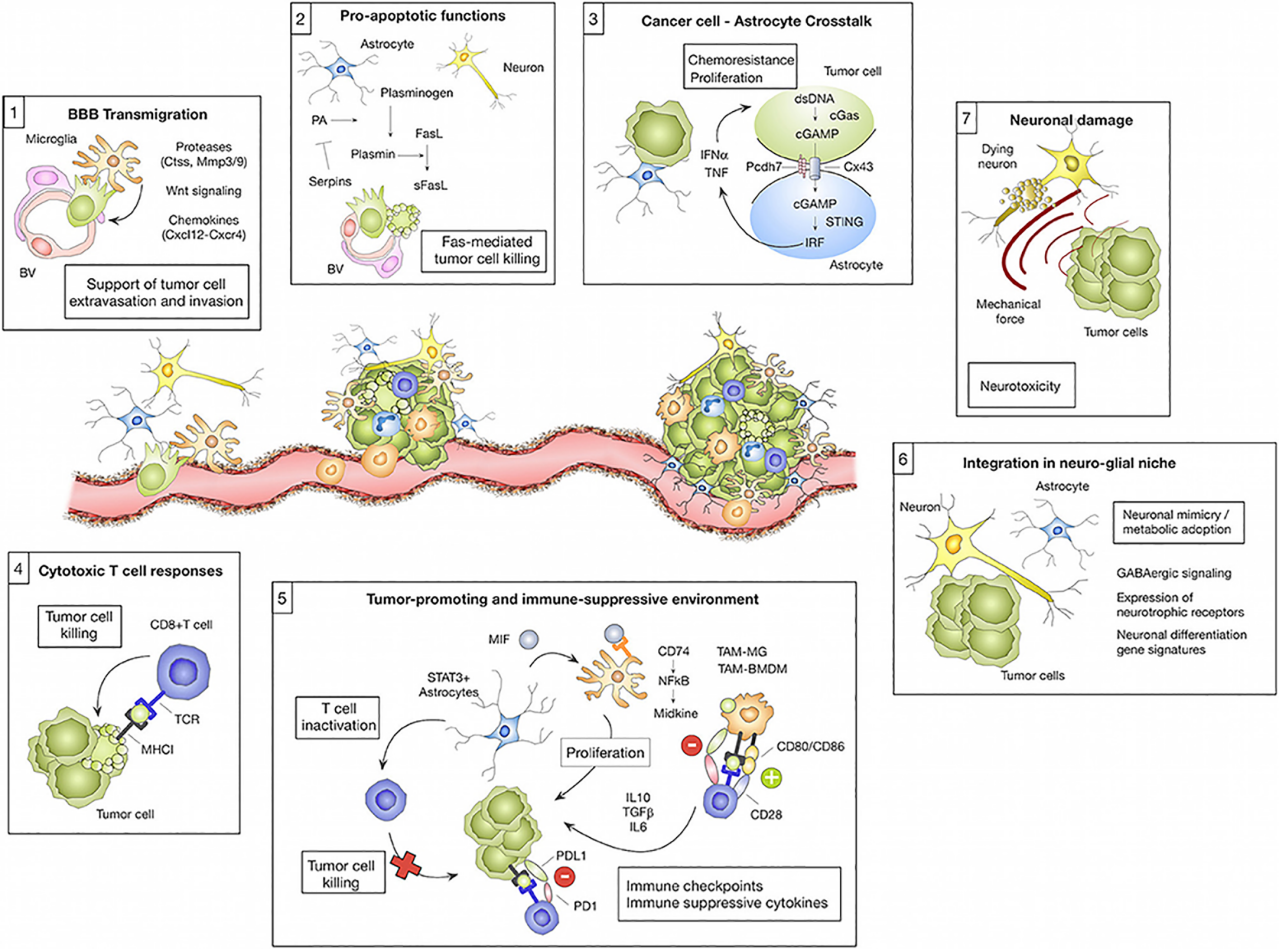
The microenvironment regulates transmission cascades. Cell types residing in the brain and those recruited from elsewhere in the body can have pro-tumor or anti-tumor effects on brain metastases depending on the cell type and the cancer stage. (1) Microglia-derived factors, such as proteases (e.g., Ctss, Mmp3, and Mmp9), Wnt regulating elements, and chemokines (e.g., Cxcl12), are implicated in facilitating tumor cell transmigration across the blood-brain barrier (BBB) and into the brain parenchyma. (2) However, astrocytes can prevent metastasis by inducing tumor cell death *via* soluble FASL. Serpin released by tumor cells can mitigate this effect by preventing the manufacture of active plasmin, which converts FasL to sFasL. Tumor cells die upon their initial contact with astrocytes, but continued contacts between the two cells, controlled by gap junctions, promote tumor cell proliferation and confer chemotherapy resistance. CGAS-STING activates IRF, which generates IFN and TNF when tumor cells and astrocytes exchange cGAMP. (4) Eliminating tumor cells, cytotoxic T cells are a crucial component of the adaptive immune system’s response against brain metastases. Tumor cells acquire neural markers that cause spherocytosis during brain colonization, allowing them to reside in glial niches. Reproduced under common creative licenses from (61).

Given the complexity of brain anatomy and the differences in the immune environment, different types of brain metastases show different evolutionary trajectories with the development of the disease (**Figure 2**).

### 5.1 How can neurons contribute to brain metastases?

Neurons are specialized cells that carry signals between neurons and are one of the most important and numerous cell types in the brain and spinal cord (62). However, little is currently known regarding their function in BrM (brain metastasis). Researchers in the field of BrM are mostly interested in studying astrocytes, microglia, and activated peripheral immune cells at the moment (63). It is generally accepted that neuronal cell loss and dysfunction are unintentional side effects of BrM progression and treatment (64). Constant neuroinflammation triggered by tumor-initiating microglia and astrocytes leads to neuronal cell loss. Myelin glial and oligodendrocyte function is similarly compromised in this tumor-responsive milieu, resulting in neurological disorders (65).

**Figure 3:**
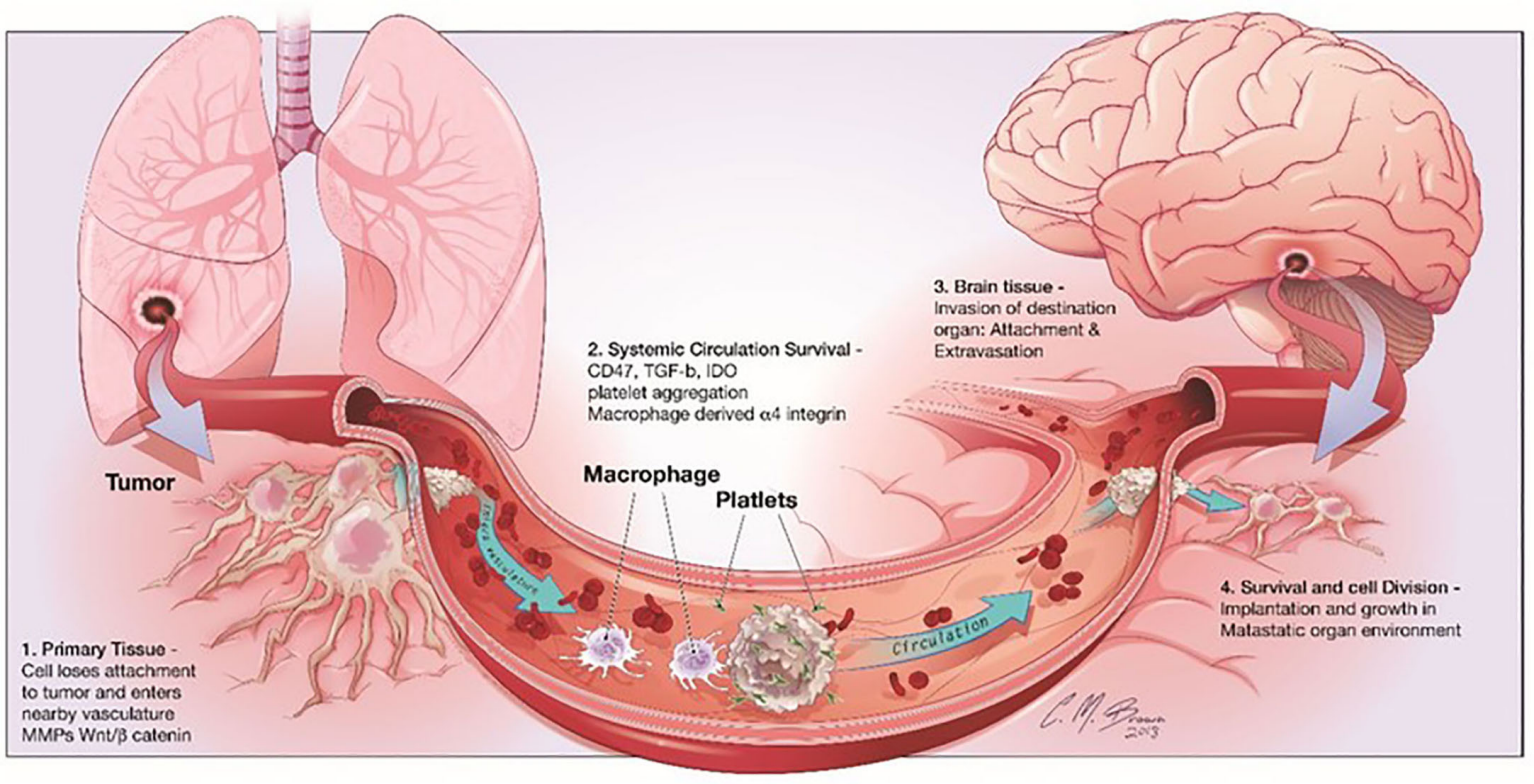
shows the stages of brain metastasis. There are four primary actions in the BMS cascade: There are four stages that metastatic cells must pass through before they may establish themselves in the CNS: 1) separation from the primary tumor, 2) surviving in the bloodstream, 3) invading the brain parenchyma, and 4) surviving in the brain’s microenvironment. Reproduced under common creative licenses from (39).

It is interesting to note that many adverse effects of chemotherapy are linked to glial dysfunction and its influence on myelin formation. The chemical brain is the term used to describe these distinctive cognitive problems (66). More recently, Seano et alinto neuronal cell death in the presence of BrM provided even more detail about the underlying mechanisms involved (67). The scientists showed that solid tumors cause indirect neural dysfunction and vascular degeneration in the peritumor region, leading to neuronal cell death through crucial distortion of the neuronal body (**Figure 3**); interestingly, the authors showed that common neuroprotective lithium medications efficiently prevent neurological damage and ameliorate some unfavorable cognitive symptoms (68). Neurons play a vital role, but their importance to cancer growth was not realized until recently. Monje et al. found that soluble extracellular NLGN3 protein related to synaptic adhesion can stimulate the PI3K pathway to promote tumor growth. As a result of these discoveries, more cancer treatments are now available. Despite breakthroughs in microfluidics devices that can detect the effects of environmental stimuli on glial cells, human astrocyte sensitivity, and myelination, few models still study the interaction between cancer cells and neurons (69). Lei et al. demonstrated through a compartmentalized microfluidic system that inhibition of nerve conduction impairs neurite support for tumor cell movement. The multiple factors that cause other organs to migrate to the brain were studied using machine learning. These methods can identify glioblastoma from a single BMS, find early tumor changes that indicate BMS, and develop new biomarkers for accurate and cost-effective detection of brain disease (70). Some *in vitro* models state the connection between nerves and cancer. Many of the mechanisms that attract metastatic cells to the brain can be studied using the organ-on-a-chip model to understand the function of the nervous system in cancer formation and development.

### 5.2 Leptomeningeal microenvironment

The pia mater is a solid monolayer of connective tissue glial and elastic fibers that lies at the very bottom of the meninges. Due to the BCSFB barrier, the CSF microenvironment is significantly cell-free, with hypoxia and nutrient deficiency (71). The low concentration of metabolic intermediates and nutrients poses a severe challenge to the growth of tumor cells. Therefore, the mechanisms of invasion, colonization, and expansion of LM are different from those of BrM, and tumor cells gradually adapt and evolve in the CSF of subarachnoid space.

Some immune cells, including monocytes, macrophages, neutrophils, and lymphocytes, were found to infiltrate the cerebrospinal fluid of LM patients (72). Scientist found that tumor cells with leptomeningeal metastasis highly expressed C3(complement component 3) and bound to the C3a receptor in choroid plexus (CP) epithelial cells (73). Amphiregulin and other mitogen nutrients are introduced into the cerebrospinal fluid circulation, disrupting the BCSFB barrier structure, which aids in the proliferation of cancer cells and improves their ability to adapt to the leptomeningeal microenvironment. Restoring the integrity of the BBB can prevent metastasis from occurring and progressing, which will provide a fresh perspective for the development of medications that target leptomeningeal metastasis (74).

### 5.3 Dural microenvironment

The dura is a nerve-immune interface containing numerous immunological cells, in contrast to the leptomeningeal environment. The dural sinus, a cerebral venous pipeline that divides the dura mater’s inner and outer layers, is crucial to the monitoring of epidemic disease (75). Recently, several reports have shown that the dura is a reservoir for the brain’s immune cells (76–78). With CSF circulation, CNS-derived antigen gathers in the vicinity of the dural sinus, is recognized by local antigen-presenting cells, and is then transmitted to the dural T cells. Immune monitoring can be facilitated by T-cell identification of CSF-derived antigens and immune cell tissues residing in the dura (79). Dural macrophages are an important part of the immune microenvironment of the dura mater. T cells can recognize antigens on the surface of dural macrophages and release chemokines. Dural macrophages are polymorphic and migrate to non-inflammatory tissues to perform immune surveillance or to inflammatory tissues to provide effectors functions (80). In addition, B lymphocytes generated in the bone marrow of the skull and vertebrae in the central nervous system can reach the dura through lymphatic ducts (81). The meningeal immunity mediated by them participates in different neuroinflammatory reactions and plays a key role in the occurrence and development of dura metastasis and other neurological diseases (82).

## 6 Microenvironmental response and tumor progression

Tumor invasion breaks the homeostasis of the central nervous system, and the microenvironment responds to foreign cells, thus affecting the progression of tumor development (83). Under the selective pressure of a new environment, a single cell or subpopulation develops into a premetastatic niche (PMN) with high spatial heterogeneity, presenting a variety of tumor cell subclasses that differ markedly from the characteristics of the primary tumor (84). Accordingly, when tumor cells initiate new adaptive mechanisms, the microenvironment system is reshaped to achieve a new state of equilibrium. In the LM animal model, CSF infiltrates many macrophages and releases proinflammatory factors IL-6 and TNF, which act on tumor cells and up-regulate the high-affinity iron transport system LCN2/SLC22A17 to “hijack” rare iron elements in the microenvironment and reduce the phagocytic function of macrophages (85). To escape immune surveillance and maintain their growth in the pia space. In the early stage of brain solid metastasis formation, PCDH7 is highly expressed in mammary adenocarcinoma and lung cancer cells. Pcdh7 interacts with astrocytes to promote the assembly of cancer-astrocyte gap junction composed of Cx43, which is used by brain metastatic cancer cells to transport cGAMP to astrocytes (86, 87). The activation of the STING pathway and its downstream STAT1 and NF-κB pathways affect the immersion and expansion of brain metastases (88). In addition, astrocytes express microRNA targeting PTEN, which is transported to tumor cells through exosomes to inhibit the expression of PTEN, thereby activating PI3K/AKT/mTOR pathway, increasing the secretion of CCL2, recruiting myeloid cells, reducing cell apoptosis, and promoting tumor growth (89).

The response mechanism of the microenvironment is closely related to tumor type. Multiple data analyses have shown that the microenvironment ecology of primary tumors, brain metastases, and gliomas is significantly different (90). The microenvironment change depends on cell subclasses’ function, location, and characteristics (91). Brain metastasis in melanoma patients is characterized by an abundance of T cells, while brain metastasis in breast cancer patients is characterized by a predominance of neutrophils (92). There are abundant macrophages and microglia in the glioma but almost no T-cell infiltration.For brain parenchymal metastases, another study used the molecular characteristics of vascular endothelial cells and parietal cells to re-annotate the blood tumor-tumor interface (BTI) formed in brain parenchymal metastases (17, 93). It is revealed that the change of functional T-cells in the microenvironment from activation to inactivation at the single-cell level is related to metabolism and microenvironment reprogramming (94). Therefore, the mechanistic analysis of microenvironmental responses in different brain tumors provides a theoretical basis and guidance for developing immunotherapies (95).

### 6.1 Interactions between tumor and stroma

Micro environmental factors, such as cell composition, division structure, hardness, tensile strain, chemotaxis, and hypoxia, primarily affect tumor growth and the ability to metastasize to the brain (50). The extracellular matrix (ECM) structurally supports cells in tissues and organs, regulating signals that influence several cell activities, including development, differentiation, and migration. ECM is composed of large molecules with specific tissue and organ components, such as glycoproteins and collagen, which regulate the mechanical properties of tissues, such as tensile strength. Surface receptors on the cell surface regulate cell activity by interacting with ECMs to produce signaling cascades. Malignancies arise from the interaction of cells with extracellular matrix components (96). Tumor cells can modify ECM structure, content, and stroma secretion to help them survive and grow. Glioma cells alter the extracellular matrix (ECM) structure of these components and the expression of cell surface receptors (97). Some malignant changes in breast and pancreatic cancer are associated with loss of the basement membrane, suggesting how altering ECM can produce conditions conducive to tumor cell invasion. The extracellular matrix in the brain regulates cellular behavior and influences tumor progression (98), so it is critical to creating physiologically accurate and suggestive *in vitro* platforms.

Brain ECMs control cell development, communication, and movement in healthy brain tissue. The ECM of the brain is dynamic, and tumor cells can change the function of various components of the brain matrix to meet their physiological needs. Aggressive malignant glioblastoma of the brain (GBM) alters the ECM of the brain to improve its viability and spread throughout the brain tissue (99). Because collagen normally supports cell movement, collagen produced by GBM increases tumor cell invasion. The main component of the brain’s ECM, hyaluronic acid (HA), is a molecule that tumor cells can alter to extend their lifespan and make them more resistant to treatment.

Several 3D bioengineering platforms, such as gelatin, have been created to assess patient-sourced brain tumor responses, combined with microenvironmental signals from underlying tumor ECMs (100). As microfluidic devices have become more sophisticated, microfluidic devices have been modified to simulate healthy and malignant tissues better using flexible extracellular matrix gels. Despite developing several biocompatible synthetic and natural biomaterials to reconstruct brain ECMs, a clearer definition of brain tissue *in vitro* and *in vivo* is still needed. This will greatly affect the way Microsystems are used to study brain diseases.

### 6.2 Neurovascular system

The vital organ bridging peripheral blood flow to the brain’s central nervous system is known as the blood-brain barrier (BBB). In addition to controlling cerebral blood flow, the neurovascular unit (NVU) maintains the very selective BBB-brain tissue balance. The neurovascular unit comprises many cell types, such as neurons, perivascular astrocytes, microglia, pericytes, endothelial cells (EC), and the basement membrane neurovascular unit (NVU). It is difficult to replicate these parts in a lab setting because of the tight connections that keep them together as a single unit. By fusing a vascular chamber with a brain chamber, the BBB and the NVU have been modelled using microfluidic devices (101). This allowed for both cellular interaction and independent hydration in the two membrane-separated regions. There have also been attempts to recreate the neurovascular environment *in vitro* using microfluidic devices containing iPSCs, with the latter yielding results that are comparable to those observed *in vivo*.

There is growing interest in replicating NVU *in vitro* because the mechanisms that lead to the deterioration of the blood-brain barrier in neurological diseases and cancers are not fully understood. Microfluid-based blood-brain barrier chip technology allows the co-culture of human stromal cells and tumor cells in a 3D extracellular matrix provided by perfusion microvessels (102). However, current limitations prevent optimal interactions between EC, pericytes, and astrocytes, essential for maintaining a favorable tumor microenvironment that largely influences cancer development. Neurovascular unit cells affect the viability of brain parenchyma but also the ability of cerebral vessels to transport metastatic tumor cells. Multiple roles of endothelial cells in brain tumor progression have been demonstrated. These include stem cell maintenance and increased therapeutic responsiveness. The role of pericytes in maintaining blood-brain barrier function has long been recognized, but only recently has it been discovered that they may also contribute to the growth of glioblastoma tumors (103). The relationship between higher pericyte concentrations in the arteries of GBM and patients’ inadequate response to chemotherapy, suggesting that these cells are potential targets for anticancer drugs. According to these studies, removing pericytes from glioblastoma increases the availability of small molecules that alter the vascular permeability of brain tumors. Understanding the biology of pericytes in the GBM microenvironment may contribute to developing more effective treatment options, as they are produced when glioma stem cells differentiate into pericytes to promote vascular development and support tumor growth. More recently, microvascular systems on a chip are beginning to meet functional requirements for assessing the dynamics of patient-derived tumors (104).

GBM tumors have a very poor prognosis for patients due to their rapid progression, invasion, and apparent resistance to current therapies. Effective distribution to tumor sites while avoiding the blood-brain barrier’s unique permeability is a significant obstacle in brain tumor treatment. The tumor’s vasculature has not changed enough to penetrate the drug effectively. The spatial and temporal distribution of drugs in blood vessels and perivascular areas can be monitored using micro-physiological platforms replicating specific functions of the human blood-brain barrier. The development of micro-NVU technology has made it possible to test potential drugs used to treat brain problems in a stable preclinical environment (105). Additional human glioblastoma samples must be made available in these microscopic tissue platforms to increase the transferability of these data to humans.

## 7 Treatments

Treating patients with brain metastases is based on systemic therapy, and the common treatment methods include surgical surgery and chemoradiotherapy (106). As mentioned above, the microenvironment response mechanism can have an important impact on the occurrence and development of brain metastases. Therefore, targeting the microenvironment is an important approach to clinical treatment (107). Immune checkpoint molecules play an increasingly prominent role in anti-tumor therapy regulating the immune microenvironment (108). Activated effector cells infiltrate tumor tissues and extensively reshape the tumor microenvironment. Clinical trials are actively carrying out comprehensive multidisciplinary treatment combined with immune checkpoint and traditional therapy (109), which prioritizes maintaining the nervous system’s normal function while also successfully inhibiting the formation of brain metastases, reducing patients’ symptoms and improving their quality of life (**Table 1**).

**Table 1:** The overview of targeted drugs and clinical progress in brain metastasis.

Targeted agent	Target	Progression-free-survival/month	Overall survival/month	Phase of trail	References
Iniparib	PARP	21.40	NA	IV	(110)
Abemaciclib	CDK4/6	6.00	22.32	II	(111)
Everolimus	P13K/Akt	>6.00	15.80	II	(112)
Veliparib	PARP	6.30	11.20	III	(113)
Lapatinib	HER2, EGFR	6.60	22.70	III	(114)
Neratinib	Her2	8.80	24.00	III	(115)
Rituximab	CD20	64.80	102.00	II	(116)
Trastuzumab	HER2	8.05	27.30	III	(117)
Dabrafenib	BRAF	7.20	24.30	II	(118)
Vemurafenib	BRAF	3.68	8.87	II	(119)
Trametinib	MEK1, MEK2	4.90	15.60	III	(120)
Osimertinib	EGFR	11.10	22.80	III	(121)
Alectinib	ALK	10.90	27.80	III	(122)
Lorlatinib	ALK, ROS1	5.60	NA	II	(123)

Source: https://clinicaltrials.gov/, NA: 95% CI (confidence interval) could not be estimated due to insufficient number of participants with response.

Despite it Widespread use of corticosteroids in cancer therapy has been shown to be particularly helpful for patients with brain cancer who have severe peritumoral edema and related neurological impairments (124). Corticosteroids have been widely used and have had a huge impact in clinical oncology over the course of several decades, but little is understood about the mechanisms by which they produce their biological and clinical effects.

### 7.1 Surgical treatment

The importance of surgical treatment for patients with brain metastases cannot be stated (125, 126). Since 1990, Scientist (127)have divided patients with brain metastases into a surgery group, whole brain radiotherapy group, surgery, and whole brain radiotherapy group according to different treatment methods and compared them. The local recurrence rate was reduced from 52% to 20% compared to the whole-brain radiotherapy group. Patients who underwent surgery and whole-brain radiation therapy saw an increase in their median survival duration from 15 to 40 weeks. With the ongoing development of diverse new techniques in the field of neurosurgery, such as functional neuroimaging, intraoperative ultrasonography, and fluorescence-guided surgery, surgical treatment has been beneficial for patients with brain metastases.

Although surgical treatment of metastatic lesions is not the standard treatment for leptomeningeal metastases, it can effectively reduce obstructive hydrocephalus and intracranial pressure according to the cerebrospinal fluid hyper pressure characteristics microenvironment (128). To treat hydrocephalus caused by leptomeningeal metastases, a ventriculoperitoneal shunt (VPS) can be performed to reduce intracranial pressure, alleviate clinical symptoms, and avoid retrograde lumbar puncture required by intrathecal chemotherapy (129, 130). Therefore, the VPS system is an effective option for patients with malignant leptomeningeal metastases, and as palliative care, it can significantly improve the quality of life of such patients. However, VPS systems in the use of a certain risk, such as infection, bleeding, and other complications should actively explore safer treatment means.

### 7.2 Radiation and chemotherapy

Radiotherapy at the symptomatic site is the standard palliative care modality, and a focused approach to the lesion is more effective in the neuro-rich parenchymal microenvironment. At present, a variety of radiotherapy regimens are available, including whole brain radiotherapy (WBRT), craniospinal radiotherapy, or focal radiotherapy to large disease areas (stereotactic irradiation) (131–133). WBRT can alleviate the neurological symptoms of patients with brain metastases and improve the local control of tumors, but it does not have a significant survival advantage (134, 135). Stereotactic irradiation is more targeted than whole-brain radiotherapy (136). However, due to its accompanying considerable bone marrow suppression, the activity of blood cell precursors in the bone marrow decreases, which affects the hematopoietic and immune functions of patients, severely limiting the application of this protocol in the treatment of chemotherapy patients (137). In addition, craniospinal irradiation (CSI) also plays an important role in the multidisciplinary treatment of brain metastases in children and adults (138). Postoperative CSI combined with chemotherapy is not only the current standard of treatment for medulloblastoma but also can be used for brain metastases spreading in cerebrospinal fluid. Advances in radiotherapy technology are breaking the stereotype of traditional radiotherapy and providing new possibilities for the remission of patients with brain metastases (139).

Intrathecal drug delivery, which allows drugs to cross the BBB to reach the leptomeningeal space, is a common method of drug delivery for leptomeningeal diseases. However, this technology is not fully mature compared with systemic drug delivery. Lumbar puncture or a surgically implanted Ommya capsule are two methods for administering intrathecal chemotherapy directly into the meningeal cavity or lumbar cisternae (140). The toxicity of intrathecal treatment of catepib, methotrexate, and cytarabine is comparable; their side symptoms, like headache, nausea, vomiting, and fever, are common sequelae of biochemical meningitis and fungal meningitis and cannot be avoided (141).

### 7.3 Immune checkpoint therapy

For a long time, the treatment options for patients with brain metastases have been limited to several traditional cancer treatment methods, such as radiotherapy, chemotherapy, and surgery, which are not specific to the pia space and thus have poor efficacy (142). Checkpoint therapy provides new treatment options for patients with brain metastases by using particular cell types in the microenvironment and precisely regulating immune mechanisms (143). T cell checkpoint receptors play a negative role in immune regulation, which can avoid excessive immunity to autoantigens. Although this negative immune regulation avoids the occurrence of inflammation, it also provides an opportunity for the tumor to escape the immune system surveillance. By blocking T cell checkpoint receptors, immune checkpoint treatment boosts the ability of T cells to kill tumors. One of the immune checkpoint treatment medications with the highest clinical usage is ipilimumab. It can successfully block CTLA-4 on T cell surfaces (cytotoxic T lymphocyte-associated antigen-4). preventing T lymphocytes from being inhibited by CTLA-4 ligand B7 (144).

In contrast, Nivolumab and Pembrolizumab inhibit programmed death protein-1 (PD-1) on the surface of T cells. Programmed death protein-ligand 1(PD-L1) is prevented from binding to programmed death protein 1(PD-1), which prevents the inhibition of T cell activity and makes T cells have the continuous killing effect (145).Durvalumab prevents the immunosuppressive effect of PD-L1/PD-1 on T cells by binding to PDL1 on T and blocking its binding to PD-1. Clinical trial data demonstrate that immune checkpoint therapy-related agents effectively treat brain metastases (146, 147). In a phase II non-randomized open-label study of pembrolizumab, patients with NSCLC and melanoma brain metastases were included. Participants in the trial included 18 people with cancer and 18 people with NSCLC (148). Two-thirds of patients and two-thirds of control patients fulfilled the RECIST(response assessment methods in solid tumors) assessment criteria, and this response was maintained throughout the follow-up period. Median survival time for patients treated with Pembrolizumab was 7.7 months, compared to only 4–6 weeks for patients with NSCLC who did not receive any treatment (149). Metastatic melanoma patients with at least one nonirradiated brain metastasis and no neurologic symptoms were given nivolumab (1 mg/kg of body weight) + ipilimumab (3 mg/kg of body weight) every 3 weeks for up to four doses, then nivolumab (3 mg/kg of body weight) every 2 weeks until progression or intolerable toxic effects in a phase 2 study. Clinical benefit was measured by the proportion of patients who achieved a complete response, partial response, or disease stabilization for at least 6 months due to treatment of intracranial tumors. In melanoma patients with untreated brain metastases, nivolumab plus ipilimumab demonstrated clinically significant intracranial effectiveness, consistent with extracranial action (150).

Although clinical trials related to treating leptomeningeal metastases have been carried out, immune microenvironment response, cell-cell interactions, and the inflammatory effects of cerebrospinal fluid and neural networks in leptomeningeal metastases are still inconclusive. In addition, the potential risk of adverse effects should be considered when administering immunotherapy for brain metastases. Immunotherapy overactivated the immune system, triggering a cytokine storm that can lead to the side effects of CNS. For example, immune checkpoint therapy may aggravate perifocal edema and increase the risk of radiation necrosis at previously exposed sites (151), and other adverse effects include intracranial hemorrhage, epilepsy, and headache (152). How to avoid the toxic and side effects of immunotherapeutic agents will also become the focus of research related to immune checkpoint therapy.

## 8 Conclusions

It is becoming better acknowledged how important the TME is to BrM. Particularly in BrM, the subject of tumor immunology is has begun to be explored. Although the brain has long been thought of as an immunological safe haven, Recent studies have revealed that BrM cause immune cells to migrate inwards from the periphery, and that antigen presentation routes exist between the brain and the rest of the body. Having both native brains and recruited cells from the periphery, BrM increases the potential for TME-targeted therapies or immunotherapies. Recently published research has begun to provide light on the intricacy of tumor-stroma interactions and heterotypic communication across niche cells that mutually control effector activities, all of which are related with BrM.

When compared to extracranial tumors, the immune milieu surrounding intracranial malignancies is markedly different and more specialized. The microenvironment of brain metastases has been extensively studied, leading to the identification and validation of certain potential targets and therapeutic approaches. The advent of cutting-edge tools like single-cell sequencing and liquids biopsy has allowed for significant progress in the study of the tumor microenvironment in recent years. From the vantage point of cell mapping, single-cell sequencing explores the cooperative operating style of cells. This is performed by identifying cell specificity and variations among small cells. The needs of studies examining tumor heterogeneity are mostly met by this method. Using a combination of studies based on interactomics, we can not only precisely define the disease depending on the type of the cell layer, but also establish the spatial and temporal diversity of the microenvironment and track the development of brain metastases from malignancies. Brain metastases must be treated specifically for their immunosuppressive properties. Considering a balance between inducing anti-tumor responses and maintaining tissue protective mechanisms is especially crucial for the brain because of its central role in managing higher cognitive functions. More in-depth study is needed to pave the way for the development of novel immunotherapeutic approaches for their management.

## Author contributions

Conceptualization, IK , SK and YL. Software, SK, MK. Validation, YL. Investigation, MD. Resources, YL. Data curation, NK. Writing—original draft preparation, SK and IK. Writing—review and editing, SH, IK, SK, MK and HS. Visualization, HS, SK, NK and MD. Supervision, YL. Project administration, YL. Funding acquisition, YL. All authors contributed to the article and approved the submitted version.
